# Errata

**DOI:** 10.1590/1980-549720230059erratum.2

**Published:** 2024-04-19

No artigo “Perfil de utilização de psicofármacos durante a pandemia de COVID-19 em Minas Gerais, Brasil”, com número de DOI: https://doi.org/10.1590/1980-549720230059.2, publicado no periódico Rev. bras. Epidemiol., v26: e230059, 2023, na página 2:

**Onde se lia:**

Em 2022, cerca de um bilhão de pessoas eram acometidas por algum transtorno mental diagnosticável em 2022, no entanto apenas uma pequena fração dispunha de cuidados eficazes, acessíveis e de qualidade^3^

**Leia-se:**

Em 2022, cerca de um bilhão de pessoas eram acometidas por algum transtorno mental diagnosticável, no entanto apenas uma pequena fração dispunha de cuidados eficazes, acessíveis e de qualidade^3^

